# Effects of high-intensity interval training versus moderate-intensity continuous training on blood pressure in patients with hypertension: A meta-analysis

**DOI:** 10.1097/MD.0000000000032246

**Published:** 2022-12-16

**Authors:** Lei Li, Xuan Liu, Fei Shen, Naxin Xu, Yun Li, Kun Xu, Junping Li, Yong Liu

**Affiliations:** a Beijing Sport University, Beijing, China.

**Keywords:** high-intensity interval training, hypertension, moderate-intensity continuous training, prehypertension

## Abstract

This meta-analysis aimed to compare the effects of high-intensity interval training (HIIT) and moderate-intensity continuous training (MICT) on blood pressure in patients with essential hypertension to explore more suitable training.

**Methods:**

PubMed, EBSCO, Cochrane Library, Web of Science, CNKI, and VIP databases were searched for randomized controlled trials published between January 2002 and November 2022. Weighted mean differences (WMDs) with 95% confidence intervals (CIs) were selected as the effect scale indices for the evaluation of the differences in post-intervention systolic blood pressure (SBP), and diastolic blood pressure (DBP), heart rate, maximum oxygen uptake (VO_2_max), and flow-mediated vasodilation. All these were compared using Review Manager 5.3 and Stata 14.0.

**Results:**

A total of 13 randomized controlled trials and 442 patients were included. The meta-analyses revealed no statistically significant differences between HIIT and MICT in improving SBP and DBP in patients with hypertension. Subgroup analyses revealed that HIIT was better than MICT in reducing SBP during daytime monitoring (WMD = −4.14, 95%CI: [−6.98, −1.30], *P < *.001). In addition, HIIT increased flow-mediated vasodilation more than MICT in hypertensive patients (WMD = 2.75, 95%CI: [0.43, 5.07], *P* = .02).

**Conclusion:**

HIIT and MICT have similar effects on the overall resting SBP and DBP in patients with hypertension and prehypertension. However, HIIT is better than MICT at reducing SBP during daytime monitoring. In addition, HIIT can improve vasodilation.

## 1. Introduction

Hypertension is one of the most prevalent chronic diseases and a major risk factor for cardiovascular disease. In China, the prevalence of hypertension and prehypertension among residents aged 18 years or older was 23.2% (≈ 244.5 million) and 41.3% (≈ 435.3 million), respectively, from 2012 to 2015.^[1,2]^ With increasing age and disease duration, patients with hypertension develop various complications, such as stroke and coronary heart disease, affecting this population’s quality of life and disease control.^[3,4]^ Consequently, aggressive early intervention is required to control the rise in blood pressure (BP).

At present, there are many ways to intervene in hypertension treatment.^[4,5]^ Exercise interventions are an effective alternative to medication for improving the BP status of hypertensive patients.^[6]^ As to many studies, moderate-intensity continuous training (MICT) has proven effective in reducing BP.^[7,8]^ Recently, high-intensity interval training (HIIT), which consists of repeated bouts of exercise and rest at varying intensities, has become a popular alternative to MICT due to its relatively high time efficiency and growing body of evidence demonstrating its effectiveness in lowering BP in people with hypertension.^[9–13]^

The meta-analysis did not find a greater advantage of HIIT over MICT in lowering resting BP in hypertensive patients.^[14,15]^ However, previous work has established that BP levels are related to exercise frequency, training period^[16]^ and circadian rhythms.^[17,18]^ Therefore, this study conducted a meta-analysis to compare the effects of HIIT and MICT on improving BP in patients with essential hypertension and develop efficient exercise programs for patients with essential hypertension.

## 2. Methods

We followed the guidelines of the Preferred Reporting Items for Systematic Reviews and Meta-Analyses (PRISMA) 2020 statement^[19]^ for the meta-analysis. The study protocol was registered at PROSPERO (CRD42022322913). Ethical approval was unnecessary because this study was based on a literature analysis.

### 2.1. Literature search strategy

Hypertension (blood pressure, high OR blood pressures, high OR high blood pressure OR high blood pressures) and high-intensity interval training (high-intensity interval trainings OR interval training, high-intensity OR interval trainings, high-intensity OR training, high-intensity interval OR trainings, high-intensity interval OR high-intensity intermittent training OR training, high-intensity intermittent OR high-intensity intermittent trainings OR trainings, high-intensity intermittent OR sprint interval training) and aerobic training (trainings, aerobic OR training training OR training trainings OR training, training OR trainings, training) were searched in PubMed, EBSCO, Cochrane library, Web of Science, CNKI, and VIP databases from January 2002 to November 2022 for randomized controlled trials (RCTs). The detailed search strategy is shown in the Table 1.

**Table 1:** Detailed search strategy.

Electronic databases	Search strategy
Cochrane	ID Search Hits #1 MeSH descriptor: [Hypertension] explode all trees #2 (Blood Pressure, High):ti,ab,kw OR (Blood Pressure, High):ti,ab,kw OR (High Blood Pressure):ti,ab,kw OR (High Blood Pressures):ti,ab,kw (Word variations have been searched) #3 #1OR#2 #4 (High Intensity Interval Training):ti,ab,kw OR (High-Intensity Interval Trainings):ti,ab,kw OR (Interval Training, High-Intensity):ti,ab,kw OR (Interval Trainings, High-Intensity):ti,ab,kw OR (Training, High-Intensity Interval):ti,ab,kw OR (Trainings, High-Intensity Interval):ti,ab,kw OR (High-Intensity Intermittent Exercise):ti,ab,kw OR (Exercise, High-Intensity Intermittent):ti,ab,kw OR (High-Intensity Intermittent Exercises):ti,ab,kw OR (Exercises, High-Intensity Intermittent):ti,ab,kw OR (Sprint Interval Training):ti,ab,kw #5 MeSH descriptor: [High-Intensity Interval Training] explode all trees #6 #4OR#5 #7 MeSH descriptor: [Exercise] explode all trees #8 (Exercises):ti,ab,kw OR (Physical Activity):ti,ab,kw OR (Activities, Physical):ti,ab,kw OR (Activity, Physical):ti,ab,kw OR (Physical Activities):ti,ab,kw OR (Exercise, Physical):ti,ab,kw OR (Exercises, Physical):ti,ab,kw OR (Physical Exercise):ti,ab,kw OR (Physical Exercises):ti,ab,kw OR (Acute Exercise):ti,ab,kw OR (Acute Exercises):ti,ab,kw OR (Exercise, Acute):ti,ab,kw OR (Exercise, Isometric):ti,ab,kw OR (Exercises, Isometric):ti,ab,kw OR (Isometric Exercises):ti,ab,kw OR (Isometric Exercise):ti,ab,kw OR (Exercise, Aerobic):ti,ab,kw OR (Aerobic Exercise):ti,ab,kw OR (Aerobic Exercises):ti,ab,kw OR (Exercises, Aerobic):ti,ab,kw OR (Exercise Training):ti,ab,kw OR (Exercise Trainings):ti,ab,kw OR (Training, Exercise):ti,ab,kw OR (Trainings, Exercise):ti,ab,kw #9 #7OR#8 #10 #6OR#9 #11 #3AND#10

### 2.2. Literature inclusion and exclusion criteria

Inclusion criteria:

(1) Study type: RCTs.
(2) Study population: adult patients with essential hypertension and prehypertension who met the diagnostic criteria for essential hypertension. The Chinese guidelines for the prevention and treatment of hypertension define prehypertension as systolic blood pressure (SBP) of 120–139 mm Hg (1 mm Hg = 0.133 kPa) and diastolic blood pressure (DBP) of 80 to 89 mm Hg during quiet time, while hypertension is defined as an SBP of ≥ 140 mm Hg and DBP ≥ 90 mm Hg during quiet time.^[20]^ In this study, prehypertension and hypertension were collectively referred to as hypertension.
(3) Intervention: Experimental group underwent HIIT, in which high-intensity exercise was performed between 80% and 100% of the peak heart rate (HR), interspersed with intervals of light exercise.^[21]^ Moreover, exercise intensity could also be defined using maximal oxygen uptake (VO_2_max), heart rate reserve (HRR), or rating of perceived exertion when the value was equal to 80% to 100% of the peak HR. The control group performed MICT consisting of moderate-intensity continuous exercise with an intensity of 50% to 70% of the peak HR. In addition, VO_2_max, HRR, or rating of perceived exertion could also be used to define exercise intensity, and values equal to 50% to 70% of the peak HR could be included.^[22]^
(4) Outcomes: The primary outcomes were post-intervention SBP and DBP, and the secondary outcomes were HR, VO_2_max, and flow-mediated vasodilation (FMD).

Exclusion criteria were as follows: non-RCTs, reviews, and animal experiments; literature for which valid data could not be extracted; continuous exercise interventions of <4 weeks; and duplicate publications.

### 2.3. Data extraction

Two authors extracted and entered the data from the included studies in an independent double-blind manner according to the needs of the study, and any disagreement was resolved by discussion. Literature extraction included data related to the first author of the literature, year of publication, sample size, gender, age of the subjects, and intervention protocol.

### 2.4. Risk of bias assessment

The methodological quality of the included literature was evaluated using Cochrane risk-of-bias tools, including random sequence generation, allocation concealment, blinding, incomplete outcome data, selective reporting, and other possible biases. Quality scoring was based on low, high, and unclear levels.

### 2.5. Grade of evidence

The quality of evidence will be evaluated following the Grading of Recommendations, Assessment, Development, and Evaluation (GRADE) approach using the GRADE profiler software. Quality is classified as high, moderate, low, or very low.

### 2.6. Statistical analysis

We calculated the agreement between the 2 reviewers regarding data screening and selection using the kappa (κ) statistic. Continuous variables are expressed as weighted mean differences (WMDs) and 95% confidence intervals (CIs). Cochran’s *Q* test combined with the *I*² test was used to determine the heterogeneity between trials. The random-effects model was used in cases of significant heterogeneity (*I*^2^ ≥ 50% and *P* ˂.1); otherwise, a fixed-effects model was used. Publication bias was investigated using funnel plot, Egger’s test and Begg’s test. Sensitivity analysis was performed using a one-by-one exclusion method to evaluate the stability of the results of this study. Significance was set at a 2-tailed *P* value of < .05. All analyses were performed using the Review Manager 5.3 and Stata 14.0.

## 3. Results

### 3.1. Basic information of the included literature

**Figure 1.**
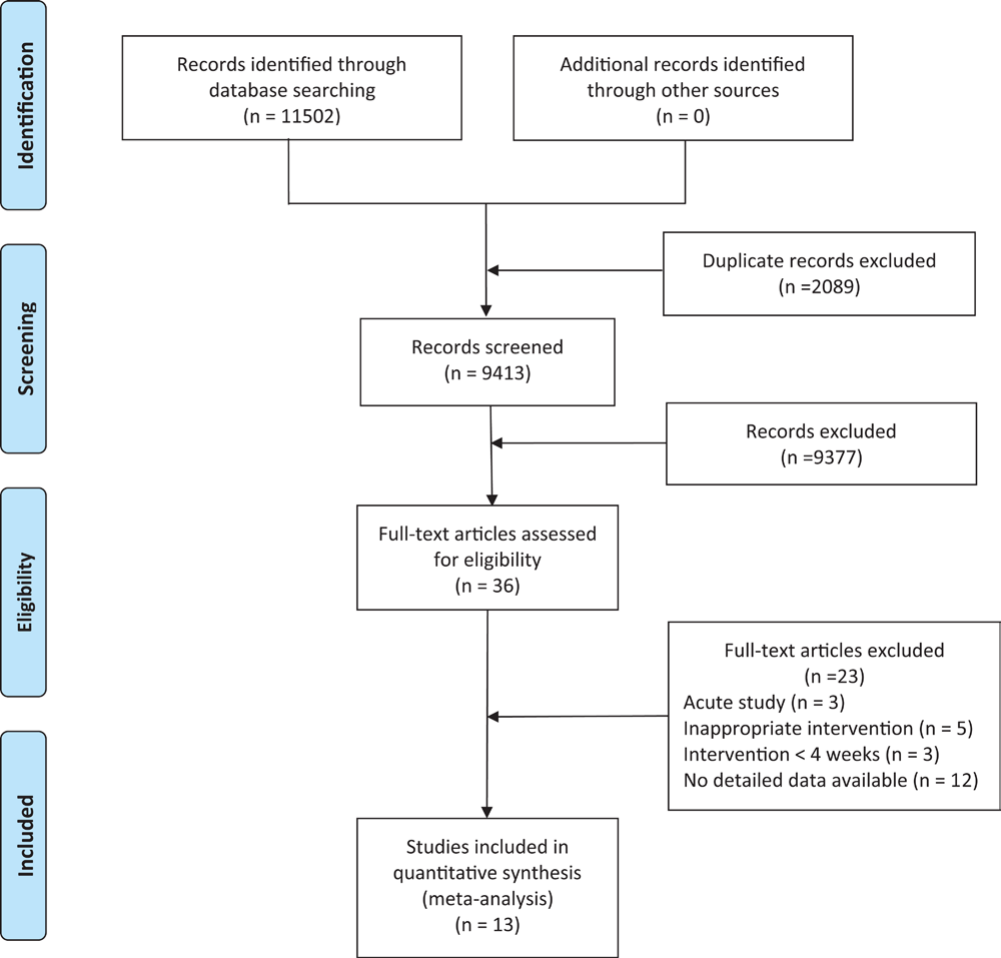
Literature screening process.

In this study, 13 articles were included in stepwise screening.^10–12,23–32^ A total of 442 patients: 224 in the HIIT and 218 in the MICT groups, were included. The literature screening process is displayed in Figure 1, the basic information of the included literature is illustrated in Table 2, and the intervention protocol of the included studies are displayed in Table 3. Agreement between the authors was good (κ = 0.83, *P* < .001).

**Table 2:** Basic characteristics of the included literature.

		HIIT				MICT			
Study	Sample (HIIT/MICT)	Age (yr)	Sample (M/F)	BMI (Kg/m²)	Baseline BP (mm Hg)	Age (yr)	Sample (M/F)	BMI (Kg/m²)	Baseline BP (mm Hg)
Ballesta-García 2020 (Spain)	17/12	66.3 ± 5.4;	0/17	30.4 ± 4.1	151.1 ± 11.7;	70.0 ± 8.8;	0/12	30.1 ± 3.08	155.6 ± 13.6;
					73.9 ± 5.0				73.9 ± 7.2
Clark 2020 (Australia)	16/12	30 ± 6;	16/0	29.0 ± 3.1	127.5 ± 8.8;	26 ± 8;	12/0	28.2 ± 2.5	130.1 ± 8.8;
					75.8 ± 6.0				72.2 ± 5.3
Cuddy 2019 (USA)	12/15	40.8 ± 10.8;	-	-	123.8 ± 6.6;	42.2 ± 9.7;	-	-	126.7 ± 14.4;
					82.2 ± 4.9				81.7 ± 7.1
Eun-Ah 2018 (Korea)	7/7	51.0 ± 10.1;	6/1	23.0 ± 1.7	125.5 ± 6;	51.6 ± 6.3;	2/5	22.9 ± 2.4	121 ± 12;
					77.1 ± 6.5				76.4 ± 8.7
Ghardashi Afousi 2018 (Iran)	18/17	54.8 ± 6.2;	9/9	29.2 ± 0.9	131.4 ± 10.4;	53.1 ± 4.8;	10/7	28.9 ± 1.0	135.7 ± 11.0;
					79.2 ± 3.0				79.9 ± 4.1
Guimarães 2010 (Brazil)	15/16	45.0 ± 9.0;	3/12	29.0 ± 5.0	123.0 ± 9.0;	50.0 ± 8.0;	7/9	28.0 ± 4.0	124.0 ± 9.0;
					78.0 ± 6.0				79.0 ± 9.0
Iellamo 2021 (Italy)	12/12	64.5 ± 7.2;	12/0	28.9 ± 1.7	122.6 ± 28.4;	66 ± 4.7;	12/0	27.9 ± 4.3	121.8 ± 33.1 ;
					81.7 ± 16.4				81.3 ± 14.6
Jo, E. A 2020 (Korea)	17/17	49.9 ± 7.3;	12/5	24.6 ± 2.8	132.1 ± 14.9;	51.8 ± 8.5;	6/11	24.4 ± 2.5	126.2 ± 11.2;
					79.3 ± 8.5				78.6 ± 7.6
Jung 2015 (Canada)	10/16	51.0 ± 11.0;	-	33.1 ± 7.7	125.0 ± 15.0;	51.0 ± 10.0;	-	32.8 ± 5.0	124.0 ± 7.0;
					79 ± 8				81.0 ± 8.0
Molmen-Hansen 2012 (Norway)	25/23	52.5 ± 7.4;	15/10	26.8 ± 4.1	141.0 ± 12.5;	53.6 ± 6.5;	13/10	27.9 ± 3.2	146.5 ± 13.1;
					85.0 ± 8.1				88.5 ± 8.4
Ramos 2016 (Australia)	15/17	57.0 ± 11.0;	10/5	31.0 ± 4.0	126.0 ± 8.0;	55.0 ± 11.0;	12/5	33.0 ± 6.0	125.0 ± 8.0;
					80.0 ± 8.0				83.0 ± 7.0
Shepherd 2015 (United Kingdom)	42/36	42.0 ± 11.0;	12/30	27.7 ± 5.0	123.0 ± 10.0;	43.0 ± 11.0;	14/22	27.7 ± 4.6	123.0 ± 13.0;
					75.0 ± 9.0				76.0 ± 9.0
Liu Xianghui 2018 (China)	18/18	52.8 ± 11.6;	10/8	26.7 ± 3.5	133.5 ± 14.5;	53.9 ± 12.2;	10/8	26.1 ± 3.1	138.2 ± 17.5;
					85.1 ± 10.1				92.2 ± 11.8

ABPM = ambulatory blood pressure monitoring, BMI = body mass index, BP = blood pressure, F = female, HIIT = high-intensity intermittent training, MICT = moderate-intensity continuous training, M = male, NR = not reported.

**Table 3:** Exercise interventions of the included literature.

Study		HIIT			MICT		
	Modality	Training modalities	Frequency (d/wk)	Duration (wk)	Training modalities	Frequency (d/wk)	Duration (wk)
Ballesta-García 2020 (Spain)	Mixed Movement	(6-12) × (1–1.5) min intervals at RPE: 16–18,	2	18	18–42 min RPE:12-14	2	18
		interspersed by 2 min active recovery at RPE: 12–14					
Clark 2020 (Australia)	Walk and Run	10 × 1 min intervals at 90%-100% HRmax,	3	6	30 min 65%–75% HRmax	3	6
		interspersed by 1 min active recovery at 15% HRmax					
Cuddy 2019 (USA)	Cycling	3 × 20 s cycling sprint.	4	8	30 min 50%–65% HRR	5	8
		Active recovery 3 min slow pedal					
Eun-Ah 2018 (Korea)	Walk and run	5 × 3 min intervals at 80% HRmax,	5	4	35 min 60% HRmax	5	4
		interspersed by 3 min active recovery at 40% HRmax					
Ghardashi Afousi 2018 (Iran)	Cycling	12 × 1.5 min intervals at 85%–90% HRmax,	3	12	42 min 70% HRmax	3	12
		interspersed by 2 min active recovery at 55-60% HRmax					
Guimarães 2010 (Brazil)	Walk and run	3 × 1 min intervals at 80% HRR,	2	16	40 min 60% HRR	2	16
		50% HRR					
Iellamo 2021 (Italy)	Walk and run	3 × 5 min intervals at 80%–95% VO_2_max,	7	12	45 min 55%–70% VO_2_max	7	12
		Active recovery 10 min low intensity exercise					
Jo, E. A 2020 (Korea)	Walk and run	5 × 3 min intervals at 80% HRR,	3	8	35 min 60% HRR	3	8
		interspersed by 3 min active recovery at 40% HRR					
Jung 2015 (Canada)	Walk and run	10 × 1 min intervals at 90% HRmax,	3	4	50 min 65% HRmax	3	4
		interspersed by 1 min active recovery at low intensity exercise					
Molmen-Hansen 2012 (Norway)	Walk and run	4 × 4 min intervals at 90%–95% HRmax,	3	12	47 min 70% HRmax	3	12
		interspersed by 3 min active recovery at 60%–70% HRmax					
Ramos 2016 (Australia)	Walk and run	4 × 4 min intervals at 85%–95% HRmax,	3	16	30 min 60%–70% HRmax	5	16
		interspersed by 3 min active recovery at 50%–70% HRmax					
Shepherd 2015 (United Kingdom)	Cycling	4 × 15–60 s intervals at 90% HRmax,	3	10	45 min 70% HRmax	3	10
		Active recovery 45–120 s					
Liu Xianghui 2018 (China)	Walk and run	1 min intervals at RPE: 18–20, interspersed by 1 min active recovery at RPE: 9–10	3	16	20 min RPE: 11–13	3	16

HIIT = high-intensity intermittent training, HRmax = maximum heart rate, HRR = heart rate reserve, MICT = moderate-intensity continuous training, RPE = rating of perceived exertion, VO_2_max = maximum oxygen uptake.

### 3.2. Methodological assessment of the included literature

**Figure 2.**
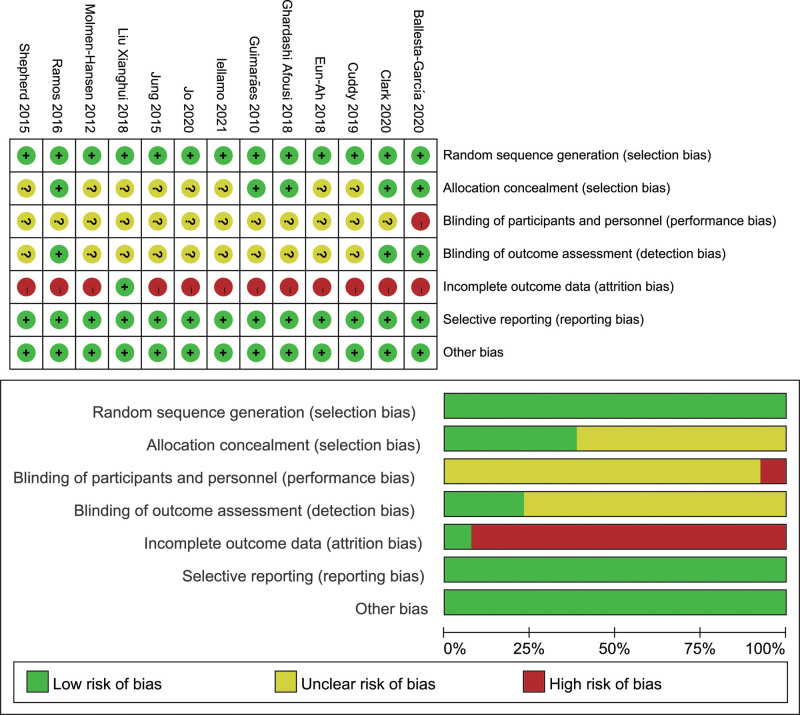
Risk of bias assessment.

The included literature was assessed methodologically (Fig. 2), and 7 were moderately risk-biased, with “+” for attainment, “−” for nonattainment, and “?” for unclear.

### 3.3. Primary outcomes

#### 3.3..1. SBP

**Figure 3.**
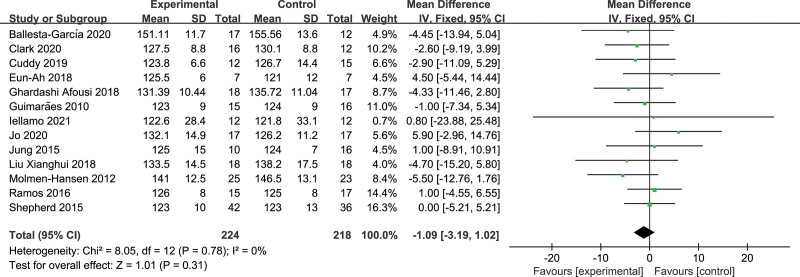
Forest plot of systolic blood pressure.

The effects of HIIT versus MICT on SBP were reported in 13 studies. There was no significant heterogeneity among the studies (*I*^2^ = 0%, *P* = .78). The result of meta-analysis demonstrated no significant difference in the anti-hypertensive effect of HIIT on SBP compared with MICT in patients with hypertension (WMD = −1.09, 95%CI: [−3.19, 1.02], *P* = .31) (Fig. 3).

Subgroup analysis was performed according to the dynamic monitoring time, and HIIT was found to be more effective in lowering SBP than MICT for daytime (WMD = −4.14, 95%CI: [−6.98, −1.30], *P < *.001) and evening monitoring (WMD = −10.26, 95%CI: [−12.65, −7.87], *P < *.001). Furthermore, subgroup analyses were also performed by BP ranges, exercise frequencies, and training cycles, but no significant differences were found in the SBP-lowering effect of HIIT compared to MICT (Table 4).

**Table 4:** Subgroup analyses of systolic blood pressure.

Outcomes	Number of studies	Sample size (HIIT/MICT)	Heterogeneity test		Value of effect		Two-tailed test	
			*I*^2^ (%)	*P*	WMD value	95%CI	Z	*P*
Dynamic monitoring time								
Daytime	4	70/69	0	.85	−4.14	[−6.98, −1.30]	2.85	.00
Evening	4	70/70	91	.0001	−10.26	[−12.65, −7.87]	8.41	.00
Blood pressure range								
Hypertension	2	42/35	0	.86	−5.24	[−11.21, 0.73]	1.72	.09
Pre-hypertension	11	182/183	0	.83	−0.47	[−2.73, 1.79]	0.40	.69
Exercise frequency								
<3 times	2	32/28	0	.55	−2.06	[−7.34, 3.21]	0.77	.44
≥3 times	11	192/190	0	.67	−0.90	[−3.20, 1.40]	0.77	.44
≥5 times	2	19/19	0	.79	3.98	[−5.24, 13.20]	0.85	.40
Training cycle								
<8 wk	3	33/35	0	.49	−0.10	[−4.90, 4.71]	0.04	.97
≥8 wk	10	191/183	0	.70	−1.32	[−3.66, 1.02]	1.11	.27
≥12 wk	7	120/115	0	.80	−2.32	[−5.23, 0.60]	1.56	.12
≥16 wk	4	65/63	0	.69	−1.09	[−4.68, 2.50]	0.59	.55

CI = confidence interval, HIIT = high-intensity intermittent training, MICT = moderate-intensity continuous training, WMD = weighted mean difference.

#### 3.3..2. DBP

**Figure 4.**
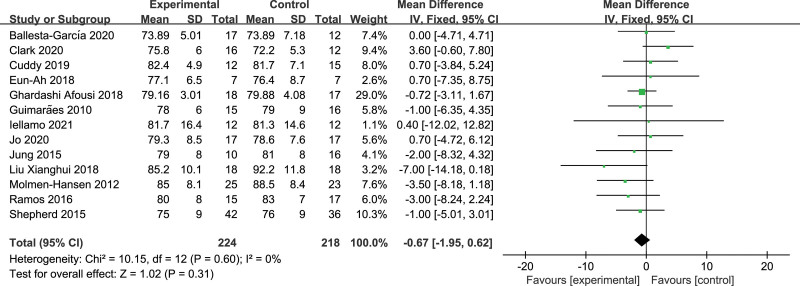
Forest plot of diastolic blood pressure.

Thirteen studies reported the effect of HIIT versus MICT on DBP; no significant heterogeneity was found among these studies (*I*^2^ = 0%, *P* = .60). The result of meta-analysis depicted no significant difference in the anti-hypertensive effect of HIIT on DBP compared with MICT in patients with hypertension (WMD = −0.67, 95%CI: [−1.95, 0.62], *P = *.31) (Fig. 4).

Further subgroup analysis demonstrated that DBP was closer to 80 mm Hg after the HIIT intervention, although HIIT was less effective in lowering DBP monitored at night than MICT (WMD = 3.63, 95%CI: [1.75, 5.51], *P < *.001). In addition, there were no significant differences in the DBP-lowering effect of HIIT compared with MICT in different BP ranges, exercise frequencies, and training cycles (Table 5).

**Table 5:** Subgroup analyses of diastolic blood pressure.

Outcomes	Number of studies	Sample size (HIIT/MICT)	Heterogeneity test		Value of effect		Two-tailed test	
			*I*^2^ (%)	*P*	WMD value	95%CI	Z	*P*
Dynamic monitoring time								
Daytime	4	70/69	29	.24	1.06	[−1.05, 3.17]	0.98	.33
Evening	4	70/70	39	.19	3.63	[1.75, 5.51]	3.78	.0002
Blood pressure range								
Pre-hypertension	7	82/85	0	.57	−2.31	[−4.65, 0.04]	1.93	.05
Normal	6	114/100	0	.73	0.04	[−1.50, 1.57]	0.05	.96
Exercise frequency								
<3 times	2	32/28	0	.78	−0.44	[−3.97, 3.10]	0.24	.81
≥3 times	11	192/190	1	.44	−0.70	[−2.08, 0.68]	1.00	.32
≥5 times	2	19/19	0	.97	0.61	[−6.14, 7.36]	0.18	.86
Training cycle								
<8 wk	3	33/35	0	.34	1.70	[−1.51, 4.90]	1.04	.30
≥8 wk	10	191/183	0	.79	−1.12	[−2.52, 0.28]	1.56	.12
≥12 wk	7	120/115	0	.65	−1.55	[−3.21, 0.11]	1.83	.07
≥16 wk	4	65/63	10	.34	−1.34	[−3.25, 0.56]	1.39	.17

CI = confidence interval, HIIT = high-intensity intermittent training, MICT = moderate-intensity continuous training, WMD = weighted mean difference.

### 3.4. Secondary outcomes

As illustrated in Table 6, no significant differences in HR and VO_2_max with HIIT compared to MICT were found, but HIIT increased FMD more than MICT in hypertensive patients (WMD = 2.75, 95%CI: [0.43, 5.07], *P* = .02).

**Table 6:** Meta-analyses of secondary outcomes.

Outcomes	Number of studies	Sample size (HIIT/MICT)	Heterogeneity test		Value of effect		Two-tailed test	
			*I*² (%)	*P*	WMD value	95%CI	Z	*P*
HR	8	151/136	0	.73	−0.66	[−2.65, 1.34]	0.65	.52
VO_2_max	4	85/86	0	.78	1.48	[−0.20, 3.16]	1.73	.08
FMD	3	49/47	0	.64	2.75	[0.43, 5.07]	2.32	.02

CI = confidence interval, FMD = flow-mediated vasodilation, HIIT = high-intensity intermittent training, HR = heart rate, MICT = moderate-intensity continuous training, VO_2_max = maximal oxygen uptake, WMD = weighted mean difference.

### 3.5. Literature publication bias and sensitivity analysis

**Figure 5.**
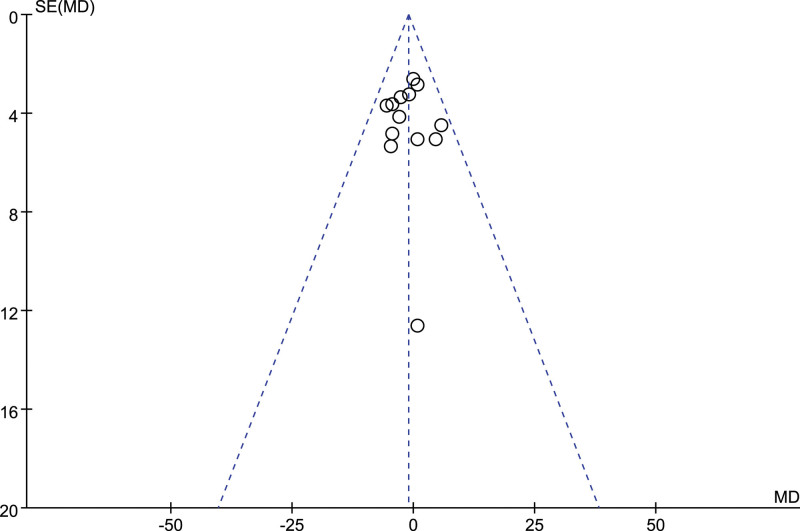
Funnel plot of systolic blood pressure.

**Figure 6.**
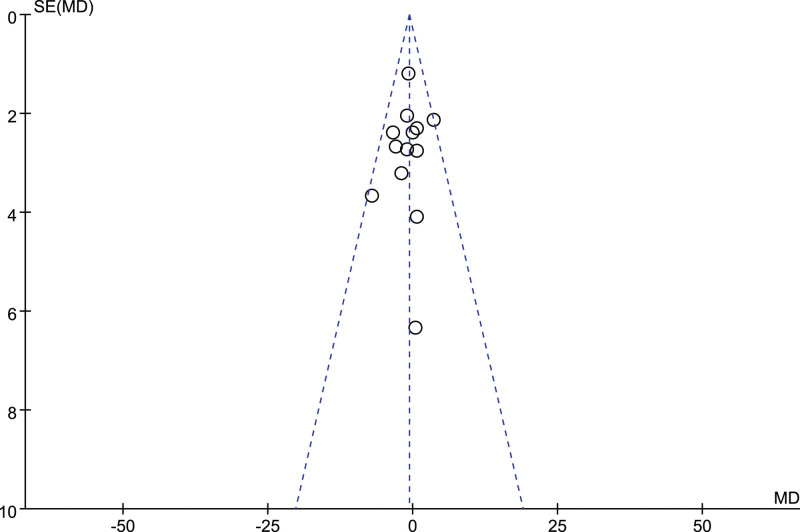
Funnel plot of diastolic blood pressure.

No visual indications of funnel plot asymmetry were observed (Figs 5 and 6). Egger’s test and Begg’s test results revealed no publication bias in SBP (Egger: *P* = .96; Begg: *P* = .95) and DBP (Egger: *P* = .59; Begg: *P* = .58). A sensitivity analysis was performed by sequentially removing each study. According to the results, no significant changes were observed for pooled WMDs and relevant 95%CIs, suggesting that all the pooled results were not influenced by any included single study, and the results of this meta-analysis were stable.

### 3.6. Grade of evidence

The quality of evidence for each eligible outcome was assessed using the GRADE approach. The results are presented in Tables 7–9. These indicated that the quality of evidence were moderate for overall SBP and DBP. All other outcomes were either of low or very low quality.

## 4. Discussion

This meta-analysis aimed to compare the effects of HIIT and MICT on BP in patients with essential hypertension. The results revealed that the pooled effect sizes of HIIT on SBP and DBP in hypertensive patients were not statistically different from those of MICT, which is consistent with the results of Costa et al.^[14]^ However, in this study, a subgroup analysis based on 24 h ambulatory BP monitoring time revealed that the effect of HIIT on daytime monitoring of SBP in patients with hypertension was better than that of MICT. Larsen et al believe that the mechanisms that guide post-exercise BP reduction are related to hemodynamic and neural factors.^33^ A meta-analysis conducted by Ramos et al^34^ inferred that HIIT has a greater positive influence on cardiorespiratory fitness and biomarkers associated with vascular function than MICT does. In addition, in the included literature, all experimental protocols were performed in the morning, affecting the activity of the sympathetic nervous system after HIIT, which may improve the vascular pressure reflex control center, resulting in lower BP during the day after exercise. Moreover, the SBP monitored at night demonstrated high heterogeneity (*I*^2^ = 91%, *P < *.001). After removing the literature Iellamo,^26^ we found a significant reduction in the heterogeneity, but the results were not statistically significant (WMD = 0.18, 95%CI: [−2.89, 3.25], *P* = .91). The reasons for this may be related to the lack of previous exercise experience, lower baseline BP, and medications taken by the patients in this study.

Regarding DBP, we did not find any significant differences between HIIT and MICT, which agrees with the results of Ciolac et al.^[14]^ However, these results are different from Ciolac et al,^14^ who presented an additional reduction of 1.2 mm Hg in DBP when HIIT was performed. In addition, these results are also different from those of Currie et al^35^: 7 mm Hg versus 2 mm Hg, which presents a favorable reduction to MICT. These differences may be due to factors such as the use of medications, diet, and lifestyle of the participants in each study.^36^

In a subgroup analysis based on BP range, the effects of HIIT and MICT on SBP and DBP were similar in both hypertensive and prehypertensive patients. However, in hypertensive patients, the effect of HIIT on SBP reduction was statistically significant (*P* = .08), suggesting that HIIT may be more effective in hypertensive patients. Furthermore, there were no significant differences in the effects of both exercise modalities on lowering SBP and DBP at different exercise frequencies and training cycles. This is consistent with previous studies showing that the hypotensive effects of intermittent and continuous exercise in the prehypertensive population were similar after the same amount of exercise.^37^ In other words, discrepancies between results may be due to differences in the exercise prescription parameters (type, intensity, and program duration). Additionally, insulin sensitivity associated with good levels of cardiorespiratory fitness depicted a significant dose effect with greater exercise volumes and higher exercise intensities, producing greater benefits.^38^ Previous studies revealed that for HIIT (12 weeks, three times per week), it seems that the interval duration should be greater than 2 min at 80% to 95% HRmax to surpass the positive effect of MICT on insulin sensitivity.^39,40^

HR is a common indicator of normal physiological function in humans, and an increase in HR contributes to the probability of death in patients with and without cardiovascular disease.^41^ Both HIIT and MICT are effective in reducing HR in hypertensive patients. The reduction in resting HR may be due to exercise-induced changes in cardiac adaptations that contribute to improved output per beat, cardiopulmonary adaptations, and cardiac autonomic function.^42^ However, in this study, no difference was found in the resting HR reduction after the 2 exercises, which may result from the identical exercise volume prescribed for both exercises.

VO_2_max is an important cardiorespiratory capacity indicator and the strongest prognostic marker of cardiovascular mortality. Enhanced VO_2_max can reduce the risk of mortality associated with cardiorespiratory disease. This study found a trend for HIIT compared with MICT in improving VO_2_max in patients with hypertension and prehypertension (*P* = .08). Previous studies have indicated that the development of VO_2_max depends on exercise intensity.^43^ Costa et al^[14]^ reported greater improvement in VO_2_max with HIIT intervention in their study. The stroke volume of the heart is a mediator of VO_2_max, which appears to result in a greater oxygen pulse after HIIT than MICT.

FMD is a vital indicator of vasodilation, and current studies suggest that exercise-induced FMD is associated with nitric oxide production by endothelial cells stimulated by arterial wall shear stress.^44^ Nitric oxide is one of the most important endogenous vasodilators in having a role in the inhibition of the aggregation of platelets, oxidative stress, vascular smooth muscle cells, recruiting of leukocytes and leukocyte adhesion.^45^ This study demonstrated that the effect of HIIT on FMD was superior to that of MICT. Haram et al^46^ demonstrated that frequent stimulation from alternating high-intensity and low-intensity exercises can produce greater shear stress on the vessel wall. Therefore, HIIT induced greater shear stress and produced more nitric oxide than MICT, contributing to vasodilation and lowered BP.

HIIT may be associated with greater safety concerns than MICT; however, a related meta-study^[14]^ found no significant adverse events in HIIT versus MICT, and HIIT was slightly better than MICT in terms of completion rates and attendance at exercise training sessions. Furthermore, the ratings of perceived enjoyment were greater after HIIT than after MICT.^47^ Overall, HIIT may be an optimal form of exercise beyond the traditional MICT.

This study revealed the effect of HIIT and MICT on BP status in patients with essential hypertension and prehypertension. However, this study has several limitations. First, hypertension prevention and treatment guidelines differ by country, and using Chinese hypertension prevention and treatment guidelines in this study may have biased the results. For instance, we did not find significant anti-hypertensive differences between HIIT and MICT in prehypertensive patients. However, according to the American Heart Association Hypertension Guidelines, an SBP of 130 to 139 mm Hg and DBP of 80 to 89 mm Hg are defined as prehypertensive.^48^ Meta-analysis of the data in the included literature that met SBP of 130 to 139 mm Hg and DBP of 80 to 89 mm Hg found that HIIT had a statistically significant lowering effect on SBP compared to MICT (WMD = −4.79, 95%CI: [−8.91, −0.66], *P* = .02). Second, the overall number of studies and the sample size of the included studies were small. Additional large-scale, high-quality studies are necessary to reach definitive conclusions. Third, the participants varied in terms of the training methods. The consistency of training methods should be considered in future meta-analyses. Finally, we only investigated the efficacy of HIIT versus MICT, but safety and patient preferences are also important considerations. Therefore, these results should be cautiously interpreted.

## 5. Conclusions

HIIT and MICT have similar effects on the overall resting SBP and DBP in patients with hypertension and prehypertension. However, HIIT is better than MICT at reducing SBP during daytime monitoring. In addition, HIIT can improve vasodilation.

**Table 7:** Systolic blood pressure and its subgroup outcomes.

Outcomes	Risk of bias	Inconsistency	Indirectness	Imprecision	Other considerations	Quality of evidence
SBP	Serious*	No serious	No serious	No serious	None	⊕⊕⊕◯ MODERATE
Subgroup of SBP						
Dynamic monitoring time						
Daytime	Serious*	No serious	No serious	Serious†	None	⊕⊕◯◯ LOW
Evening	Serious*	Serious‡	No serious	Serious†	None	⊕◯◯◯ VERY LOW
Blood pressure range						
Hypertension	Serious*	No serious	No serious	Serious†	None	⊕⊕◯◯ LOW
Pre-hypertension	Serious*	No serious	No serious	No serious	None	⊕⊕⊕◯ MODERATE
Exercise frequency						
<3 times	No serious	No serious	No serious	Serious†	None	⊕⊕⊕◯ MODERATE
≥3 times	Serious*	No serious	No serious	No serious	None	⊕⊕⊕◯ MODERATE
≥5 times	Serious*	No serious	No serious	Serious†	None	⊕⊕◯◯ LOW
Training cycle						
<8 wk	No serious	No serious	No serious	Serious†	None	⊕⊕⊕◯ MODERATE
≥8 wk	Serious*	No serious	No serious	No serious	None	⊕⊕⊕◯ MODERATE
≥12 wk	Serious*	No serious	No serious	Serious†	None	⊕⊕◯◯ LOW
≥16 wk	Serious*	No serious	No serious	Serious†	None	⊕⊕◯◯ LOW

GRADE Working Group grades of evidence.

**High certainty**: We are very confident that the true effect lies close to that of the estimate of the effect.

**Moderate certainty**: We are moderately confident in the effect estimate: The true effect is likely to be close to the estimate of the effect, but there is a possibility that it is substantially different.

**Low certainty**: Our confidence in the effect estimate is limited: The true effect may be substantially different from the estimate of the effect.

**Very low certainty**: We have very little confidence in the effect estimate: The true effect is likely to be substantially different from the estimate of effect.

* Incomplete outcome data.

† The sample size is small.

‡ Results were highly heterogeneous across included studies.

**Table 8:** Diastolic blood pressure and its subgroup outcomes.

Outcomes	Risk of bias	Inconsistency	Indirectness	Imprecision	Other considerations	Quality of evidence
DBP	Serious*	No serious	No serious	No serious	None	⊕⊕⊕◯ MODERATE
Subgroup of DBP						
**Dynamic monitoring time**						
Daytime	Serious*	Serious‡	No serious	Serious†	None	⊕◯◯◯ VERY LOW
Evening	Serious*	Serious‡	No serious	Serious†	None	⊕◯◯◯ VERY LOW
**Blood pressure range**						
Hypertension	Serious*	No serious	No serious	Serious†	None	⊕⊕◯◯ LOW
Pre-hypertension	Serious*	No serious	No serious	Serious†	None	⊕⊕◯◯ LOW
**Exercise frequency**						
<3 times	No serious	No serious	No serious	Serious†	None	⊕⊕⊕◯ MODERATE
≥3 times	Serious*	Serious‡	No serious	No serious	None	⊕⊕◯◯ LOW
≥5 times	Serious*	No serious	no serious	Serious†	None	⊕⊕◯◯ LOW
**Training cycle**						
<8 wk	No serious	No serious	no serious	Serious†	None	⊕⊕⊕◯ MODERATE
≥8 wk	Serious*	No serious	No serious	No serious	None	⊕⊕⊕◯ MODERATE
≥12 wk	Serious*	No serious	No serious	Serious†	None	⊕⊕◯◯ LOW
≥16 wk	Serious*	Serious‡	No serious	Serious†	None	⊕◯◯◯ VERY LOW

GRADE Working Group grades of evidence.

**High certainty**: We are very confident that the true effect lies close to that of the estimate of the effect.

**Moderate certainty**: We are moderately confident in the effect estimate: The true effect is likely to be close to the estimate of the effect, but there is a possibility that it is substantially different.

**Low certainty**: Our confidence in the effect estimate is limited: The true effect may be substantially different from the estimate of the effect.

**Very low certainty**: We have very little confidence in the effect estimate: The true effect is likely to be substantially different from the estimate of effect.

DBP = diastolic blood pressure.

* Incomplete outcome data.

† The sample size is small.

‡ Results were highly heterogeneous across included studies.

**Table 9:** Secondary outcomes.

Outcomes	Risk of bias	Inconsistency	Indirectness	Imprecision	Other considerations	Quality of evidence
HR	Serious*	No serious	No serious	Serious†	None	⊕⊕◯◯ LOW
VO_2_max	Serious*	No serious	No serious	Serious†	None	⊕⊕◯◯ LOW
FMD	Serious*	No serious	No serious	Serious†	None	⊕⊕◯◯ LOW

GRADE Working Group grades of evidence.

**High certainty**: We are very confident that the true effect lies close to that of the estimate of the effect.

**Moderate certainty**: We are moderately confident in the effect estimate: The true effect is likely to be close to the estimate of the effect, but there is a possibility that it is substantially different.

**Low certainty**: Our confidence in the effect estimate is limited: The true effect may be substantially different from the estimate of the effect.

**Very low certainty**: We have very little confidence in the effect estimate: The true effect is likely to be substantially different from the estimate of effect.

FMD = flow-mediated vasodilation, SBP = systolic blood pressure, VO2max = maximum oxygen uptake.

* Incomplete outcome data.

† The sample size is small.

## Acknowledgements

Thank you very much to my parents and my wife, Xuan Liu, who gave me strength everywhere.

## Author contributions

**Conceptualization:** Lei Li, Yong Liu.

**Data curation:** Lei Li, Fei Shen, Naxin Xu, Yun Li, Kun Xu.

**Formal analysis:** Lei Li, Fei Shen, Naxin Xu, Yun Li, Kun Xu.

**Investigation:** Lei Li, Xuan Liu.

**Methodology:** Lei Li, Xuan Liu.

**Project administration:** Lei Li, Xuan Liu.

**Resources:** Lei Li, Xuan Liu, Yong Liu.

**Software:** Lei Li, Xuan Liu.

**Supervision:** Xuan Liu, Yong Liu.

**Writing – original draft:** Lei Li.

**Writing – review & editing:** Xuan Liu, Junping Li, Yong Liu.
